# Alpha-lipoic acid ameliorates sodium valproate-induced liver injury in mice

**DOI:** 10.14202/vetworld.2020.963-966

**Published:** 2020-05-22

**Authors:** Chrismawan Ardianto, Hijrawati Ayu Wardani, Nurrahmi Nurrahmi, Mahardian Rahmadi, Junaidi Khotib

**Affiliations:** Department of Clinical Pharmacy, Faculty of Pharmacy, Universitas Airlangga, Surabaya 60115, Indonesia

**Keywords:** alpha-lipoic acid, drug-induced liver injury, histopathological, liver injury, sodium valproate

## Abstract

**Aim:**

This study examines the effect of alpha-lipoic acid (ALA) on sodium valproate-induced liver injury through histological features of mice liver tissue.

**Materials and Methods:**

Mice were divided into three groups; (1) vehicle group, (2) sodium valproate group, and (3) sodium valproate-ALA group. The vehicle group was injected with saline intraperitoneal (i.p.) for 28 days. The sodium valproate group was injected with sodium valproate 300 mg/kg, i.p. daily for 2 weeks, after which the vehicle was administered daily until day 28. The sodium valproate-ALA group was injected with sodium valproate 300 mg/kg daily for 2 weeks before the administration of ALA 100 mg/kg i.p. until day 28. The mice were euthanized, and the liver was extracted for histopathological examination.

**Results:**

Histopathological examination of the liver section of the vehicle group showed a normal structure of the liver. Two weeks after the administration of sodium valproate, histopathological examination showed an abnormal structure of the liver, with necrotic appearance and inflammatory cells. Moreover, treatment with ALA after the administration of sodium valproate notably ameliorated hepatic histopathological lesions and the liver structure corresponded to a normal liver structure.

**Conclusion:**

ALA ameliorates sodium valproate-induced liver injury in mice.

## Introduction

Epilepsy is one of the most common chronic neurologic disorders. Approximately 70 million people have epilepsy worldwide and approximately 90% of them are from developing regions [1]. Sodium valproate is a commonly prescribed antiepileptic drug used to treat various seizure disorders. Furthermore, severe side effects such as hepatotoxicity, pancreatitis, thrombocytopenia, and platelet aggregation are associated with valproate treatment [2]. The liver is the primary organ for the metabolism of many antiepileptic drugs and is subjected to drug-induced injury. The mechanism of hepatotoxicity remains unclear, and overproduction of reactive oxygen species (ROS) and compromised antioxidant capacity as a result of oxidative stress has been hypothesized to play a role in the etiology of toxicity. In addition, several studies have reported that valproate treatment is associated with oxidative stress [3].

However, efforts are being made to overcome drug-induced liver injury (DILI). To overcome the potential harmful effects of free radicals and to reduce the damage by oxidants, many antioxidants have been examined in trials as scavengers to stop the injury. Alpha-lipoic acid (ALA), one of the most effective antioxidants, is known to be involved in the cellular antioxidant system. ALA, known as thioctic acid or 1, 2-dithiolane-3-pentanoic acid (C_8_H_14_O_2_S_2_), is an essential cofactor in mitochondrial dehydrogenase reactions, soluble in water and lipid, and widely distributed in the cellular membrane, cytosol, and extracellular space [4]. Several investigations have reported the hepatoprotective effects of ALA [5-7]; however, there is a lack of information regarding the role of ALA in sodium valproate-induced liver injury.

This study examines the effect of ALA on sodium valproate-induced liver injury in mice observed through the histological features of the liver.

## Materials and Methods

### Ethical approval

All experiments were conducted at the Animal Research Laboratory of the Faculty of Pharmacy Universitas Airlangga, Surabaya, Indonesia, in accordance with the Guidelines for the Care and Use of Laboratory Animals issued by the National Institutes of Health revised in 1985. The Ethics Committee of Faculty of Veterinary Medicine Universitas Airlangga, Surabaya, Indonesia, approved the study protocol.

### Materials

Sodium valproate powder was obtained from the Kalbe Farma pharmaceutical industry, Indonesia. ALA was obtained from Simex Pharmaceutical Indonesia in the form of a powder. Sodium valproate 300 mg/kg was diluted in a saline solution, and ALA 100 mg/kg was diluted in 50% propylene glycol solution.

### Animals, experimental design, and treatments

Male ICR mice weighing between 25 and 30 g were used. The animals were housed in chip-bedded plastic cages at room temperature (25°C±2°C) in a 12-h light/dark cycle at the Animal Research Laboratory of the Faculty of Pharmacy Universitas Airlangga. Free access to drinking water and standard chow food was provided to the mice until the end of the study. The mice were divided into three groups; (1) vehicle group, (2) sodium valproate group, and (3) sodium valproate-ALA group. The vehicle group was injected with saline intraperitoneal (i.p.) for 28 days. The sodium valproate group was injected with sodium valproate 300 mg/kg i.p. daily for 2 weeks, after which the vehicle was administered daily until day 28. The sodium valproate-ALA group was injected with sodium valproate 300 mg/kg daily for 2 weeks, before the administration of ALA 100 mg/kg i.p. until day 28.

### Histopathological examination

At the end of the study, the mice were euthanized and the liver was extracted. Liver fragments were fixed in formalin at 10% and processed and embedded in paraffin. Further, 3 mm sections were made and subjected to hematoxylin and eosin staining. Under the optic microscope, the slides were examined and digital images were captured. The portal area, which is the most sensitive area in liver damage, was examined. The pattern of hepatocytes, infiltration of the inflammatory cells, and cell necrosis were observed.

## Results

**Figure-1:**
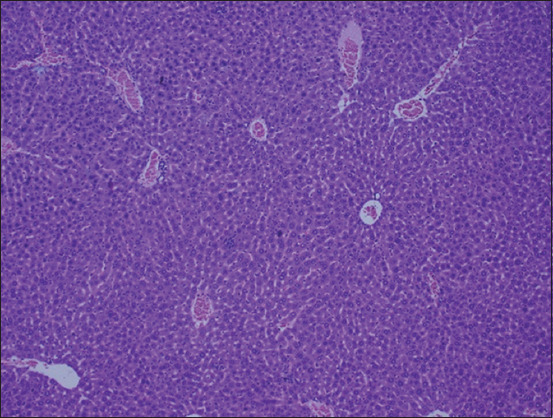
The representative figure of liver section showed normal structure in the vehicle group (100×).

**Figure-2:**
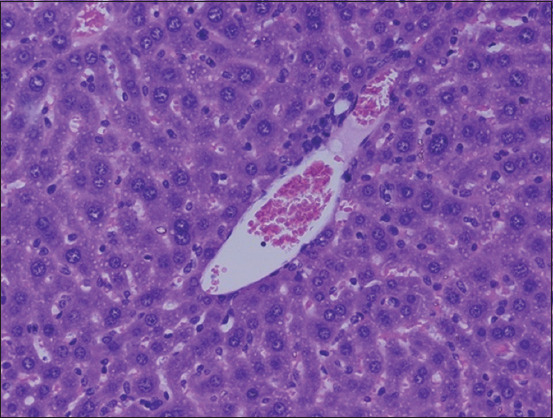
The representative figure of liver section showed normal structure in the vehicle group (400×).

**Figure-3:**
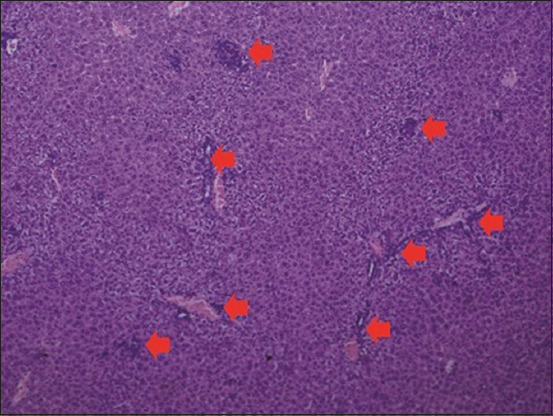
The representative figure of liver section showed abnormal structure in the sodium valproate group (100×). Necrosis and inflammatory cells infiltration were observed.

**Figure-4:**
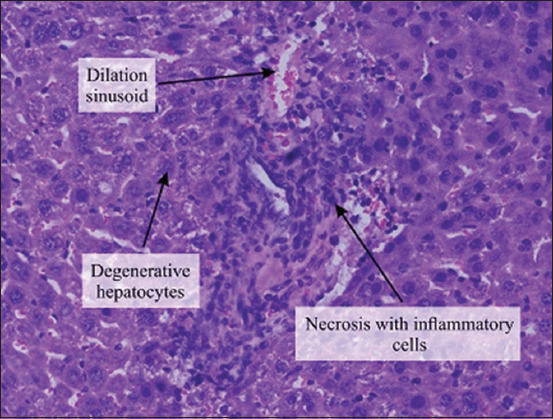
The representative figure of liver section showed abnormal structure in the sodium valproate group (400×). Necrosis with inflammatory cells infiltration, dilation of sinusoid, and degenerative hepatocytes were observed.

**Figure-5:**
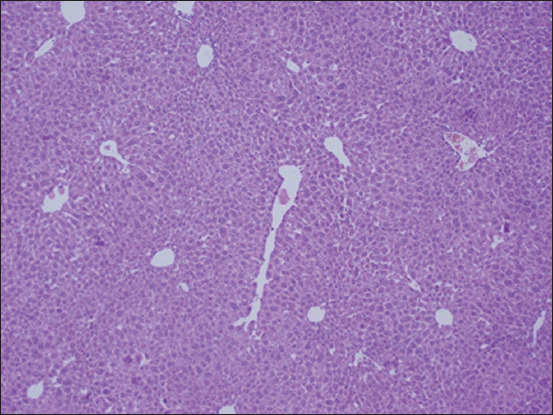
The representative figure of liver section showed normal structure in the sodium valproate-alpha-lipoic acid group (100×).

**Figure-6:**
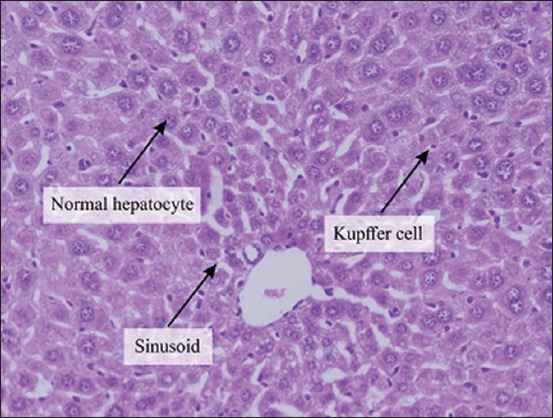
The representative figure of liver section showed normal structure in the sodium valproate-alpha-lipoic acid group (400×).

The histopathological examination of the liver section of the vehicle group showed a normal structure of the liver observed at 100× and 400× (Figures-1 and 2). Two weeks after the administration of sodium valproate, histopathological assessment exhibited an abnormal liver structure at 100× (Figure-3) and 400× (Figure-4). In the liver architecture, there were partial distortions, accompanied by focal vacuolar degenerative changes in hepatocytes. The focal areas of necrosis with the inflammatory cells were detected. In addition, the scattered focal aggregates of the inflammatory cells were observed in the portal areas and the area between hepatocytes. Moreover, the result showed mild degeneration of hepatocytes followed by widening of sinusoids and increase in Kupffer cells. Besides, the majority of the hepatocytes showed vacuolation, accompanied by variation in the size and shape of the nucleus. Furthermore, hypertrophied nuclei were observed. Treatment with ALA 100 mg/kg considerably ameliorated the hepatic histopathological lesions observed at 100× (Figure-5). Moreover, our findings showed that the liver structure corresponded to a normal liver structure observed at 400× (Figure-6).

## Discussion

Sodium valproate is known to cause DILI. The clinical feature of DILI has been reported to exhibit a specific pattern in clinical data and liver histology. Hepatocyte necrosis followed by marked inflammatory activity is the most common pattern observed in DILI and has become the gold standard in evaluating the condition [4]. The histopathological examination of the liver sections in the present study showed that the vehicle group showed a normal structure of the liver. However, the sodium valproate group exhibits impairment in several areas of the liver. Histopathological features after daily administration of sodium valproate 300 mg/kg i.p. for 2 weeks showed an abnormal structure of the liver. The sodium valproate group exhibited necrosis followed by the aggregation of the inflammatory cells, mild degeneration of hepatocytes with widening of sinusoids, and increase in Kupffer cells. The increase in Kupffer cells reflected the increase in the inflammatory process; however, the pathogenesis of such hepatotoxicity remains unclear. Several mechanisms have been proposed for valproate-induced hepatotoxicity, including reactive metabolites of valproate [8,9]. In addition, the involvement of carnitine deficiency [10,11], hyperammonemia [5], and oxidative stress or enhanced production of ROS has been reported [3,12]. This is supported by our findings showing that there was a considerable increase in serum ALT levels in the sodium valproate group compared with the vehicle group but not in serum AST levels (data not shown). ALT has been considered a reliable and sensitive marker of liver disease, and elevated serum ALT levels can effectively identify an ongoing liver disease [13].

The present investigation showed that treatment with ALA 100 mg/kg after sodium valproate-induced liver injury for 2 weeks notably decreases the hepatic lesions. The histological features showed that ALA preserved the normal structure of the liver under valproate administration. The previous investigations reported a hepatoprotective effect of ALA [5-7]. A study reported that ALA protects hepatocytes by suppressing hepatic oxidative stress as well as downregulating the expression of hepatic pro-inflammatory cytokines, iNOS, and NF-kB [14]. However, one study reported that in *in vivo*, lipoic acid was most likely associated with the inhibition of b-oxidation or glucuronidation, the two dominant metabolic processes of valproate [15]. Further studies are needed to clarify this issue.

## Conclusion

The present results indicated that ALA ameliorated sodium valproate-induced liver injury in mice. For the 1^st^ time, the present study provided direct evidence of the *in vivo* efficacy of ALA in the treatment of valproate-induced liver injury. Further research is needed to clarify the protective mechanism of ALA in valproate-induced liver injury and the prospects of ALA in clinical use for such indication.

## Authors’ Contributions

CA, HAW, and JK designed the plan of work. CA, HAW, MR, and NN performed laboratory investigation. CA, HAW, MR, and JK participated in draft and revision of the manuscript. All authors read and approved the final manuscript.

## References

[1] Singh A, Trevick S (2016). The epidemiology of global epilepsy. Neurol. Clin.

[2] Ibrahim M.A (2012). Evaluation of hepatotoxicity of valproic acid in albino mice, histological and histochemical studies. Life Sci. J.

[3] Tong V, Teng X.W, Chang T.K.H, Abbott F.S (2005). Valproic acid I:Time course of lipid peroxidation biomarkers, liver toxicity, and valproic acid metabolite levels in rats. Toxicol. Sci.

[4] Kleiner D.E (2017). Drug-induced liver injury:The hepatic pathologist's approach. Gastroenterol. Clin. North Am.

[5] Cattaneo C.I, Ressico F, Valsesia R, D'Innella P, Ballabio M, Fornaro M (2017). Sudden valproate-induced hyperammonemia managed with L-carnitine in a medically healthy bipolar patient:Essential review of the literature and case report. Med(United States).

[6] Al-Rasheed N.M, Fadda L, Al-Rasheed N.M, Hasan I.H, Ali H.M, Mohamad R.A (2017). Hepatoprotective role of a-lipoic acid and thymoquinone in acetaminophen induced liver injury:Down-regulation of COX-2 and flt-1 expression. Braz. Arch. Biol. Technol.

[7] Phua L.C, New L.S, Goh C.W, Neo A.H, Browne E.R, Chan E.C.Y (2008). Investigation of the drug-drug interaction between a-lipoic acid and valproate via mitochondrial b-oxidation. Pharm. Res.

[8] Zimmerman H.J, Ishak K.G (1982). Valproate-induced hepatic injury:Analyses of 23 fatal cases. Hepatology.

[9] Ghodke-Puranika Y, Thornd C.F, Lamba J.K, Leederf J.S, Song W, Birnbaum A.K, Altmand R.B, Klein T.E (2013). Valproic acid pathway:Pharmacokinetics and pharmacodynamics. Pharmacogenet. Genomics.

[10] Coulter D.L (1981). Carnitine deficiency:A possible mechanism for valproate hepatotoxicity. Lancet.

[11] Li Q, Song W, Jin H (2018). Carnitine deficiency in Chinese children with epilepsy on valproate monotherapy. Indian Pediatr.

[12] Tung E.W.Y, Winn L.M (2011). Valproic acid increases formation of reactive oxygen species and induces apoptosis in postimplantation embryos:A role for oxidative stress in valproic acid-induced neural tube defects. Mol. Pharmacol.

[13] Kim W.R, Flamm S.L, Di Bisceglie A.M, Bodenheimer H.C (2008). Serum activity of alanine aminotransferase (ALT) as an indicator of health and disease. Hepatology.

[14] Sadek K.M, Saleh E.A, Nasr S.M (2018). Molecular hepatoprotective effects of lipoic acid against carbon tetrachloride-induced liver fibrosis in rats:Hepatoprotection at molecular level. Hum. Exp. Toxicol.

[15] Abdulrazzaq A.M, Badr M, Gammoh O, Abu Khalil A.A, Ghanim B.Y, Alhussainy T.M, Qinna N.A (2019). Hepatoprotective actions of ascorbic acid, alpha-lipoic acid and silymarin or their combination against acetaminophen-induced hepatotoxicity in rats. Medicina(Kaunas).

